# On the Acquisition of High-Quality Digital Images and Extraction of Effective Color Information for Soil Water Content Testing

**DOI:** 10.3390/s22093130

**Published:** 2022-04-20

**Authors:** Guanshi Liu, Shengkui Tian, Yankun Mo, Ruyi Chen, Qingsong Zhao

**Affiliations:** 1 State Key Laboratory of Geomechanics and Geotechnical Engineering, Institute of Rock and Soil Mechanics, Chinese Academy of Sciences, Wuhan 430071, China; gsliu@whrsm.ac.cn; 2 Guangxi Key Laboratory of Rock and Soil Mechanics and Engineering, Guilin University of Technology, Guilin 541004, China; moyankun77@163.com (Y.M.); zgchenruyi@163.com (R.C.); zhaoqingsong528@163.com (Q.Z.)

**Keywords:** soil color, water content, color space, characteristic parameters, RGB

## Abstract

Soil water content (SWC) is a critical indicator for engineering construction, crop production, and the hydrologic cycle. The rapid and accurate assessment of SWC is of great importance. At present, digital images are becoming increasingly popular in environmental monitoring and soil property analysis owing to the advantages of non-destructiveness, cheapness, and high-efficiency. However, the capture of high-quality digital image and effective color information acquisition is challenging. For this reason, a photographic platform with an integrated experimental structure configuration was designed to yield high-quality soil images. The detrimental parameters of the platform including type and intensity of the light source and the camera shooting angle were determined after systematic exploration. A new method based on Gaussian fitting gray histogram for extracting RGB image feature parameters was proposed and validated. The correlation between 21 characteristic parameters of five color spaces (RGB, HLS, CIEXYZ, CIELAB, and CIELUV) and SWC was investigated. The model for the relationship between characteristic parameters and SWC was constructed by using least squares regression (LSR), stepwise regression (STR), and partial least squares regression (PLSR). Findings showed that the camera platform equipped with 45° illumination D65 light source, 90° shooting angle, 1900~2500 lx surface illumination, and operating at ambient temperature difference of 5 °C could produce highly reproducible and stable soil color information. The effects of image scale had a great influence on color feature extraction. The entire area of soil image, i.e., 3,000,000 pixels, was chosen in conjunction with a new method for obtaining color features, which is beneficial to eliminate the interference of uneven lightness and micro-topography of soil samples. For the five color spaces and related 21 characteristic parameters, RGB and CIEXYZ spaces and characteristic parameter of lightness both exhibited the strongest correlation with SWC. The PLSR model based on soil specimen images ID had an excellent predictive accuracy and the best stability (R^2^ = 0.999, RMSE = 0.236). This study showed the potential of the application of color information of digital images to predict SWC in agriculture and geotechnical engineering.

## 1. Introduction

Soil water content (SWC), one of the key parameters for research in agriculture, geotechnical engineering, and environmental science, normally with high spatio-temporal variability, has great influences on various soil properties such as hydraulic conductivity, compressibility, shear strength, and infiltration capacity [1,2]. Therefore, accurate and real-time monitoring of SWC is of great significance to crop yields, construction safety, and the hydrological cycle process [3,4,5,6,7,8].

There are numerous methods for determining SWC such as the oven-dried method, TDR (time-domain reflectometry), neutron meter for discrete point’s measurement, and the remote sensing method for large-scale monitoring of dynamic changes [3,9,10]. Among them, the oven-dried method is considered the standard due to its accuracy [4], but it consumes energy and time as it requires steps including sampling, drying, and weighting. TDR has high installation requirements, complex operation, and is vulnerable to salt, cracks, and other interference [4]. The neutron probe has a broad range of measurements and reliable accuracy, but the presence of radioactive sources is potentially hazardous to health and the environment [11]. The remote sensing method is heavily dependent on climate and vegetation cover [3]. The advantages and drawbacks of each method are detailed in Table 1. With the accelerating trend of intellectualization and unmanned operation, traditional methods are no longer suitable for massive acquisition and management of SWC data, so it is imperative to explore simpler and faster methods of determining SWC. Digital cameras have received high attention in proximal soil sensors and environmental science with the advantages of low cost, convenience, and high resolution. Studies have shown that the color of soil digital images gradually became darker with increasing moisture, so soil digital images have the potential to be a novel indirect method for predicting SWC [12,13,14].

The development of the high-resolution camera including digital cameras, smartphones, and those of image processing software has opened up another cheap approach for quantitative acquisition and analysis of soil color information. This overcomes the subjectivity of visual comparison (Munsell color charts, MCCs) and addresses the problem of high cost and tedious operation of the spectrometer [15,16]. Studies on this topic have been performed by many scholars. For example, Gómez-Robledo et al. [16] realized the digital recording and analysis of soil color by using a smartphone under controllable lighting conditions; Fan et al. [17] used smartphones to obtain the soil color under sunny and cloudy light, and the measurement accuracy was the same as that obtained by MCCs, confirming the importance of light sources for obtaining soil color; Yang et al. [18] employed the halogen lamp as a light source and a smartphone to collect soil images, so as to predict the content of soil organic matter (SOM); Fu et al. [19] predicted SOM under varying moisture based on the color parameters derived from soil image, quantified the effect of water content on the accuracy of the SOM prediction model, and concluded that the influence of water content on the accuracy of the SOM prediction model was significant when the water content exceeded 10%; Swetha et al. [20] utilized LED lights to illuminate the darkroom and the soil image obtained by a smartphone to realize rapid soil texture classification; Han et al. [21] proposed a soil classification method using RGB signals of soil images collected by smartphones and auxiliary accessories under a light source of ring LED; Heil et al. [22] used the digital camera to obtain soil images under LED lighting and developed a microscale map of iron oxides and organic carbon content based on the color parameters CIE a* and HSV V. Persson [11] analyzed the soil color in RGB and HSV space and found that there was a high correlation between parameters S, V, and water content, then established a reliable SWC estimation model with color information; Yoshimoto et al. [23] found that the relationship between saturation and image color brightness could be expressed by a quadratic function with high fitting degree, and also proposed a method to obtain a large-area soil saturation contour map by image processing; Zanetti et al. [24] applied artificial neural network (ANN) to describe the relationship between tropical soil image and water content; dos Santos et al. [13] established a linear relationship between median values of R (red), G (green), and B (blue) bands in RGB color space of soil images and SWCs, and accordingly, the non-destructive testing of SWC was realized; Gadi et al. [25] proposed a reliable quartic polynomial model for describing the relationship between the mean gray value of soil image and SWC; Belfort et al. [26] drew a high-resolution map of water content for unsaturated porous media by analysis of digital images; Lu et al. [27] utilized UAVs (unmanned aerial vehicles) to collect the visible spectrum image of steppe surface and found that the brightness value of images was significantly related to water content and the topsoil moisture could be estimated by the brightness value of UAV-visible images combined with vegetation coverage.

The above-mentioned studies confirmed that by establishing models between soil image parameters and soil attributes (moisture content, organic matter, iron oxide, etc.), we can not only obtain soil attribute information quantitatively and quickly but also realize digital mapping [18,19,22,23,26,27]. However, at present for the research on methods of water content prediction by soil color information, some aspects have not been investigated, such as how to control the main equipment parameters (intensity and type of light source) so as to obtain stable and high-quality digital images and how to extract effective color information. This results in low prediction accuracy and few practical applications in geotechnical engineering; soil tends to be darker in response to increased moisture but becomes lighter again at the highest water content; thus, linear models are unable to fully capture and interpret the complex relationship between soil color and water content.

Therefore, to obtain high-quality digital images of soil and extract effective color information for SWC testing, in this study, (1) an equipment with an integrated experimental structure configuration for collecting high-quality soil images was designed, and comparative tests were conducted to identify the core parameters of the equipment, including the type and intensity of light source and the shooting angle. (2) A new method based on Gaussian distribution fitting to extract characteristic parameters was proposed, and its feasibility in soil image processing was verified. (3) The piecewise models for water content prediction were developed from the variables related to water content selected by correlation analysis from 21 characteristic parameters.

## 2. Materials and Methods

### 2.1. Soil Sampling and Preparation

**Figure 1:**
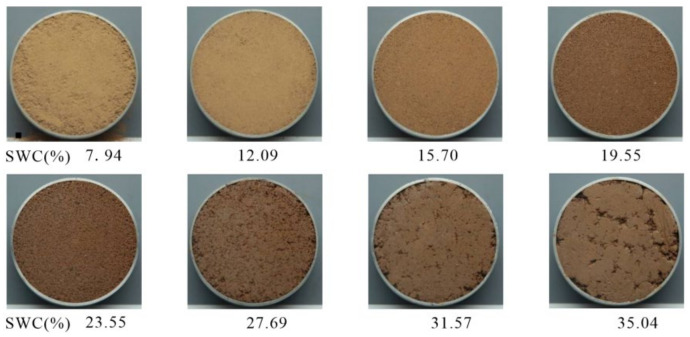
Digital images of expansive soil with different water contents.

Two types of soil for calibration and experimentation were used. One was montmorillonite of calcium (Soil 1a) and sodium (Soil 1b) with a grayish white color, homogeneous texture, and strong reflection, while the latter was collected from Nanyang, Henan Province China (32°59′23′′ N, 112°31′42′′ E). To avoid the interference of organic matter, the superficial dark brown culivated soil (about 1 m in depth) was removed before sampling, and the second layer of yellowish brown expansive soil (Soil 2, about 1~2 m in depth) was the mainly studied soil sample. Samples were air-dried, ground, and passed through a 2 mm sieve to remove impurities such as roots and large particles of soil-like loess concretion. Soil 1a and Soil 1b were packed into two ring knives (61.8 mm diameter, 2 mm height) as calibration specimens with an even surface. Similarly, a portion of Soil 2 at a known initial water content was taken and mixed with a calculated amount of distilled water by a sprayer, then target water contents could be reached, ranging from 0% to 36% in 1% increments. Four groups of parallel samples, 31 in each group and 124 in total, were prepared. All the specimens were rapidly wrapped with cling film after being removed without touching the surfaces, then put into the moisturizing cabinet for storage. Some digital images of the soil with different water contents are shown in Figure 1.

### 2.2. Photographic Parameters

#### 2.2.1. Equipment and Platform for Photography

**Figure 2:**
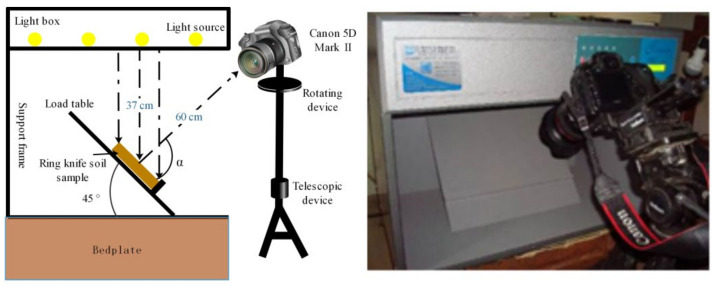
Testing apparatus.

In order to obtain high quality images of soil specimens, a high-resolution Canon camera 5D Mark II with less differences between channels was employed, which could more accurately reflect the ratio between the channels [28], and its CMOS image sensors could be primarily used for visible range (typically from 400~700 nm wavelength band) of the electromagnetic spectrums [29]. Some related parameters are summarized in Table 2. The color of same object acquired by a camera in different shooting environments can be significantly different [30] because the image sensor is not capable of correctly distinguishing color under any light intensity. To avoid this uncertainty, a test device shown in Figure 2 was designed, which consisted of four main components: box with replaceable light source, load table, support frame, and camera. The load table, a rough background plate, was fixed at a 45° angle. The light source was mounted approximately 37 cm above soil specimens, and the camera was also adjusted to a distance of about 60 cm from soil specimens. After being approximately measured using a tape, the distances were kept unchanged (Figure 2). All tests were conducted in a dark room to ensure that all light signals received by the camera were from the box.

#### 2.2.2. Type and Intensity of Light Source

To select an optimal light source, a comparison test was performed. Three kinds of common artificial light sources with same color temperature (6500 K) and power (18 W), i.e., standard light source (D65), ordinary fluorescent tubes, and LED bulbs (recorded as L1, L2, and L3, respectively) were compared. In order to check the reproducibility and stability of the three light sources for the soil color collected by the digital camera, the spatial positions of all the components were fixed. Before and after the replacement of light source, the distance and angle between the light source and the soil specimen were kept unchanged. Calibration specimen was used for light source determination. Under the illumination by the three light sources, each specimen was photographed for four times with three consecutive shots each time. After shooting, the specimen was sealed to keep moisture. The comparison test lasted for 2 h.

An intensity test was performed. D65 source, in agreement with the standard ASTM (2008) for the MCCs determination [31], with different powers was used to achieve different light intensities. To quantify the change in light intensity more intuitively, illuminance of specimen surface was used to evaluate the light intensity change. The illuminance was measured by a photometer (Taiwan TES-1330A) with a range of 20,000 lx and a minimum resolution of 0.01 lx. For each soil specimen, digital images with 10 different illuminance levels could be obtained.

#### 2.2.3. Shooting Angle and Ambient Temperature

When the distance between the camera lens and the surface of soil specimen is fixed, the angle *α* between the center line of lens and the surface of soil specimen also affects the collection of soil color information. According to bidirectional reflectance distribution function (BRDF), the reflectance is related to the angle of the signal received by the sensor [32]. Due to the limitation of experimental setup, only two angles of 45° and 90° were chosen for the comparative test since they were easy to modify and retain stability. The shooting angle was adjusted by a telescopic and rotating device to keep constant the light intensity on the surface of soil specimen. During the test, the lens plane was adjusted to be parallel to the surface of the calibration specimen. When the center line of the lens and the center of the specimen coincided, an image of the calibration specimen with *α* 90° was captured. Adjusting *α* to 45°, the above procedures were repeated for another image.

The ambient temperature is also a variable that affects the acquisition of soil color information. Fixing the device parameters and spatial location, images of calibrated soil specimens were captured at different temperatures (range: 17.60~29.80 °C, gradient: ~1 °C).

### 2.3. Image Acquisition and Optimal Representative Area

Before the soil specimen image was taken, the light source was preheated for about 15 min, then the photography platform was assembled, and *α* was adjusted to 90°. The camera was set up according to the parameters in Table 2. A gray card of X-Rite was used for the camera’s white balance. The specimen to be photographed was placed on a fixed position on the load table, and each soil specimen was photographed three times, with a total number of 372 (31 × 4 × 3) images. After image collection, the specimens were dried and weighed for SWC measurement.

Since soil is a heterogeneous material, its image must have size and space effect. The worse the surface homogeneity of a specimen is, the greater the influence on the characteristic parameters acquired from soil images is. In order to eliminate this uncertainty as much as possible, two images with a large difference in surface homogeneity of soil specimens were chosen and the sampling radius was increased, with a total of 27 grades. Simultaneously, statistical parameters including the mean and variance of image color for different sampling areas were calculated. The relationship between the sampling areas and their statistical parameters was analyzed and shown in a curve. Two areas related to the stability points at which the curves tends to be smooth could be identified, and the larger area could be determined as the optimal sampling area.

### 2.4. Image Processing and Characteristic Parameter Extraction

**Figure 3:**
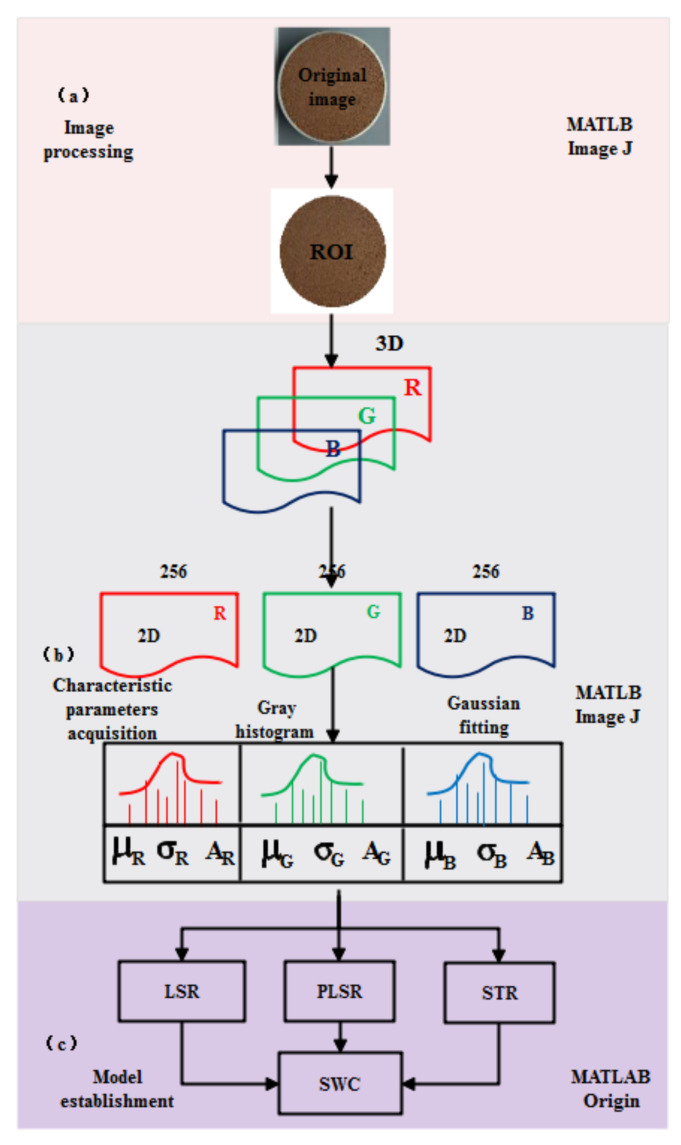
Flow chart: (**a**) image processing; (**b**) characteristic parameter acquisition; (**c**) model construction.

The procedure for image processing is shown in Figure 3a. MATLAB (Math Works, USA, 2019b version) and Image J based on java (National Institutes of Health, USA) is shown in detail in Figure 3. The hardware was a Dell computer with Intel(R) Core(TM) i7-11700 @ 2.50GHz CPU, 16.0 GB RAM, NVIDIA GeForce GT 730 and running under Windows 10 64-bit. A rectangular region (2050 × 2050 pixel) was cut from the center of the original image, and threshold segmentation was applied to extract the global information of the soil specimen. The remaining useless area of the image was masked with a white color (R = G = B = 255). After that, the effective soil color information of the image was about 3,000,000 pixels. The process of characteristic parameter extraction is shown in Figure 3b. In the process of sample preparation, there is non-soil information including holes and shadows on the surface of soil specimens. Although the non-soil information occupies a small part of the image, the distribution is discrete (Figure 1). Therefore, a simple method to obtain the characteristic parameters by fitting image gray histogram of each RGB channel with Gaussian distribution (Equation (1)) was proposed. Firstly, the RGB components of three images of same water content were separated, which were later averaged to produce an average value of each component under this water content. This could eliminate the camera jitter and inherent noise interference from the image sensor. Finally, Equation (1) was used to fit gray histogram of RGB components of image one by one.

(1) $$f\left(x\right)\;=\;A\exp\left(-{\left(x\;-\;\mu \right)}^{2}/2\sigma^{2}\right)\;+\;\beta$$

where *μ*, *σ*, and *A* are the mean, variance, and fitting parameters of Gaussian distribution, respectively. Each parameter contains three channels of RGB, namely *μ_R_*, *μ_G_*, and *μ_B_*, *σ_R_*, *σ_G_*, and *σ_B_*, and *A_R_*, *A_G_*, and *A_B_*. Among them, the three fitting parameters were divided by 2050 (pixel size along the row or column in the cut images mentioned above) for normalization to avoid the influence of image size. *μ* and *σ* are independent of each other, representing the overall distribution and dispersion of soil color, respectively. Furthermore, the auxiliary features *σ* and *A* avoid the phenomenon in which a soil image has different characteristic parameters or different soil images have the same characteristic parameters. Nine characteristic parameters are used as the ID of an image of a soil specimen, which can realize the unification of characteristic parameters of different soil specimen images. In order to contain the information of *R*, *G*, and *B* bands, equal weighted gray value ((*G_v_* = *R* + *G* + *B*)/3) as a characteristic parameter has been applied in studies [7,25], and the variations in the sensitivity of different wavelength spectra to changes in water content was not taken into account. However, prior studies have shown that the sensitivity of the *R* band to water content is the strongest, followed by the *G* band and the *B* band [13,33]. Therefore, following the conversion method of RGB space to XYZ space, different weights were given to *R*, *G*, and *B* when *μ_M_* was calculated by Equation (2).

(2) $$\mu_{M}\;=\;\left(3\mu_{R}\;+\;2\mu_{G}\;+\;\mu_{B}\right)$$

where the weight of *R*, *G*, and *B* was set as 1/2, 1/3, and 1/6, respectively, according to the appropriate descending rate of *R*, *G*, and *B* with increasing water content in steep falling section. As the RGB color space is device-dependent, it was converted to the device-independent CIE family (CIELAB, CIELUV, and CIEXYZ) and the Munsell system (HLS). To distinguish the three L (lightness, range from black to white) components in the CIELAB, CIELUV, and HLS, they were denoted as *L-AB*, *L-UV*, and *L-HS*, respectively. The other parameters were *A* (chroma, redness (positive a*) or greenness (negative a*)), *B* (chrome, yellowness (positive b*) or blueness (negative b*)), *U* (chroma, redness (positive u*) or greenness (negative u*)), *V* (chroma, yellowness (positive v*) or blueness (negative v*)), *X* (color information), *Y* (lightness), *Z* (color information), *H* (hue), and *S* (saturation). More details about the above conversion between different color spaces and the parameter definition can be found in studies [34,35]. A total of 21 characteristic parameters were extracted from the five color spaces, which were classified into four categories: hue, saturation, lightness, and auxiliary parameters. For the convenience of subsequent modeling, all image IDs were stored in a matrix *E_i_* (Equation (3)), and the water content was also incorporated into a vector *Θ_i_* (Equation (4)).

(3) $$E_{i}\;=\;\left[\begin{array}{ccc} \mu_{Ri} & \sigma_{Ri} & A_{Ri}\\ \mu_{Gi} & \sigma_{Gi} & A_{Gi}\\ \mu_{Bi} & \sigma_{Bi} & A_{Bi} \end{array}\right]\;(i\;=\;1,2,3\ldots n)$$

(4) $$\Theta_{i}\;=\;\theta_{i}\;(i\;=\;1,2,3\ldots n)$$

where and *n* is the number of samples.

**Figure 4:**
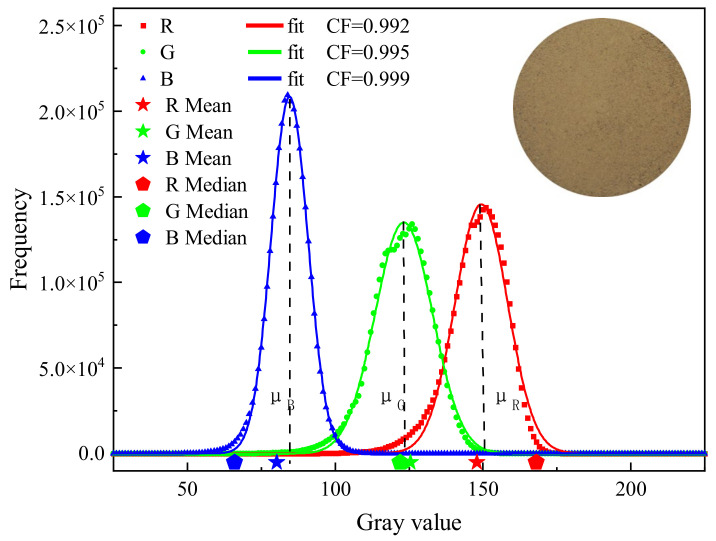
Gaussian fitting of RGB components.

**Figure 5:**
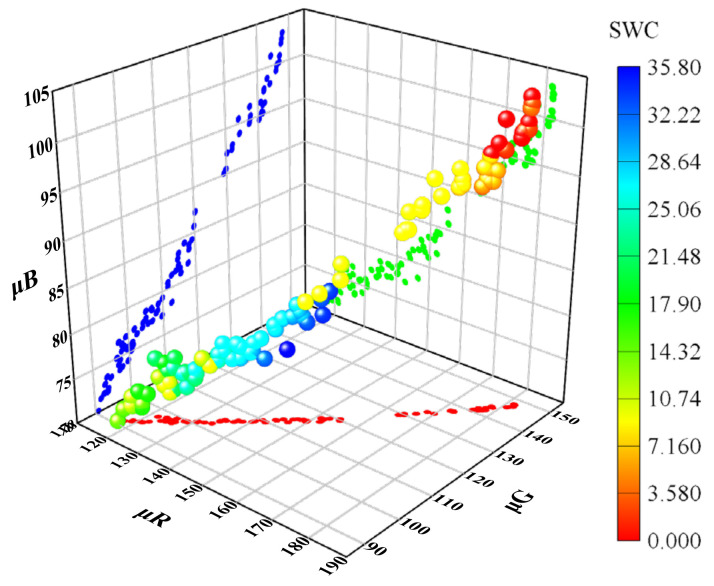
Relationship between characteristic parameters from the RGB color space and SWC.

The Gaussian fitting of RGB channels is shown in Figure 4, which shows that *μ* with statistical significance has the capacity to resist external disturbance than the mean value and the median value, and shows more representativeness. The curves of characteristic parameters from the RGB color space versus water content is shown in Figure 5, which also confirms a strong stability of the characteristic parameters. To distinguish the applicability of the 21 characteristic parameters to SWC prediction, the best prediction variables were screened by correlation analysis.

### 2.5. Modeling and Evaluation

The model construction is presented in Figure 3c. The full sets of 124 samples were randomly divided into 75% training sets (*n* = 92) and 25% validation sets (*n* = 32). Three sets had the following statistical characteristics: for the full sets, the SWC mean was 16.08%, the standard deviation (SD) 10.61, and the range 35.71%; for the training sets, the mean was 15.97%, the SD 10.18, and the range 35.71%; for the validation sets, the mean was 16.72%, the SD 10.87, and the range 34.66%. The sets had approximate distributions, which could minimize the effect of database deviation on the model [36]. In the correlation analysis, the characteristic parameters with high correlation coefficients were selected to establish the SWC prediction models of least squares regression (LSR), stepwise regression (STR), and partial least squares regression (PLSR). STR is based on the probability of the F-test to select the input variables. PLSR linearly combines input variables. STR and PLSR can both effectively avoid the effects of input variable collinearity and redundancy on the model [37,38]. To quantitatively evaluate the performance of a model, R^2^ and RMSE (Equation (5)) were used as the indexes.

(5) $$\mathrm{RMSE}\;=\;\sqrt{\frac{1}{n}\overset{n}{\underset{i\;=\;1}{\sum }}\left(y_{p}\;-\;y_{m}\right)}$$

where *y_p_* is the predicted value, *y_m_* is the measured value, and *n* is the number of samples. The closer R^2^ tends to 1, the more reliable a model is, and the more RMSE tends to 0, the higher the accuracy of the model is [39,40].

## 3. Results and Discussion

### 3.1. Sampling Area of Image Analysis

**Figure 6:**
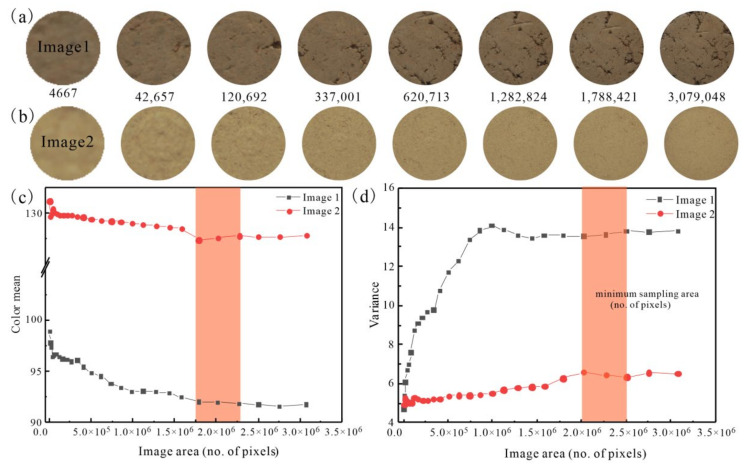
Color mean and variance of different sampling areas: (**a**,**b**) Image 1 and Image 2 with different sampling area respectively; (**c**,**d**) changes of mean and variance of color with sampling area change respectively.

Figure 6c,d shows the RGB mean and variance of two images of Soil 2 specimen (Image 1 and Image 2) with different sampling areas. Despite the fact that they have the same degree of compaction, the surface of two specimens exhibited different surface conditions owing to sample processing. Compared to Image 2, Image 1 was darker and showed more obvious holes randomly distributed on the surface, resulting in a smaller RGB mean value and larger variance. As the sampling area increased, the RGB mean of the 2 images decreased first and became steady later, while the RGB variance increased first then became steady (Figure 6c,d). It was evident that a minimum representative area exists for color information extraction of soil image. Specifically, the RGB mean became stable when the sampling area was greater than 1,750,000 pixels. Furthermore, when the sampling area was greater than 2,000,000 pixels, the variance was approximately stable. Therefore, if the sampling area was greater than 2,000,000 pixels, the statistical parameters derived from different sampling areas for Images 1 and 2 could maintain constant level around 91, 127, 13, and 6, respectively. In this case, the extracted characteristic parameters could effectively avoid the influence of image size and also eliminate the influence of brightness inconsistency caused by instrument assembly error. The minimum sampling area depends on the soil type and preparation method. The minimum sampling area for these tested soil specimens was about 2,000,000 pixels, close to the whole region of specimen. Since the image pixels were not so large that the efficiency of image data processing was not affected, the whole region (3,000,000 pixels) of a specimen was selected as the optimal sampling area if no partition processing was needed.

### 3.2. Influence of Light Source 

#### 3.2.1. Reproducibility

**Figure 7:**
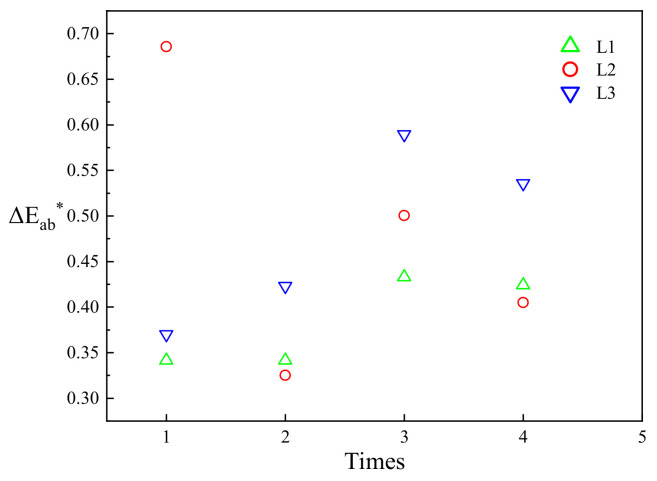
Color difference (ΔE_ab_*) of calibration soil specimen under three light sources.

To quantitatively analyze the change of soil color photographed at different times under same condition, the color difference (ΔEab*) calculated by Equation (6) [41] was introduced. It is an important index to measure the similarity between colors.

(6) $$\Delta E_{ab}{}^{*}\;=\;sqrt({\left(\Delta L^{*}\right)}^{2}\;+\;({\left(\Delta a^{*}\right)}^{2})\;+\;({\left(\Delta b^{*}\right)}^{2}))$$

where ΔL*, Δa*, and Δb* are the difference of measured color parameters from the corresponding parameters at different times. Figure 7 shows the scatter diagram of four ΔEab* of calibrated specimen under the radiation of three light sources. Overall, the ΔEab* of calibration specimen under the illumination of three light sources was less than three [42]. Among them, the ΔEab* under L1 illumination was less than 0.5, and that under L2 and L3 illumination was larger, as well as the dispersion of data. The order of ΔEab* was L1 < L2 < L3. In contrast, the color reproducibility and accuracy of digital image of calibration specimen under L1 illumination was the highest.

#### 3.2.2. Stability

**Figure 8:**
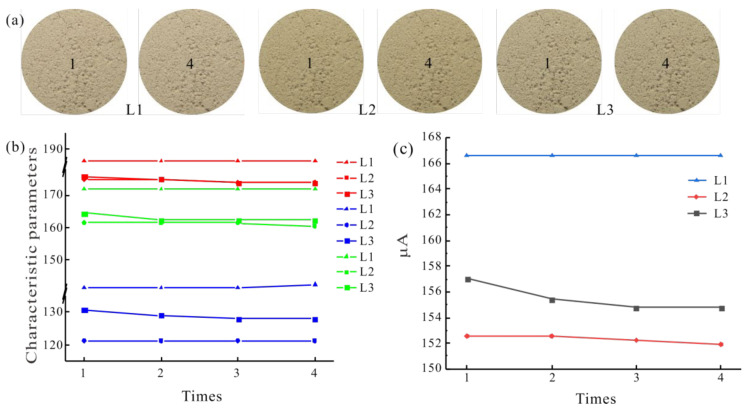
Characteristic parameters change of Soil 1b image at different times: (**a**) original images; (**b**) change of characteristic parameters from RGB color space; (**c**) *μ_A_* change.

The images of Soil 1b taken under the three light sources at different times are shown in Figure 8a. The image under L1 illumination was brighter, that under L2 illumination was darker, and that under L3 illumination was in the middle. The change of characteristic parameters from the RGB color space can be seen in Figure 8b. The RG component of the image illumination by L1 did not change, and the *B* component fluctuated slightly at the fourth time. Under L2 illumination, the RGB components fluctuated irregularly. Under L3 illumination, the RG components fluctuated a little, and the *B* component remained unchanged. The *μ_A_* ((*μ_R_* + *μ_G_* + *μ_B_*)/3)) had a similar result (Figure 8c). Therefore, the order of image stability of soil specimen captured under the three light sources for long time was L1 > L2 > L3. Comprehensively, L1 was the best light source.

#### 3.2.3. Optimal Illuminance

**Figure 9:**
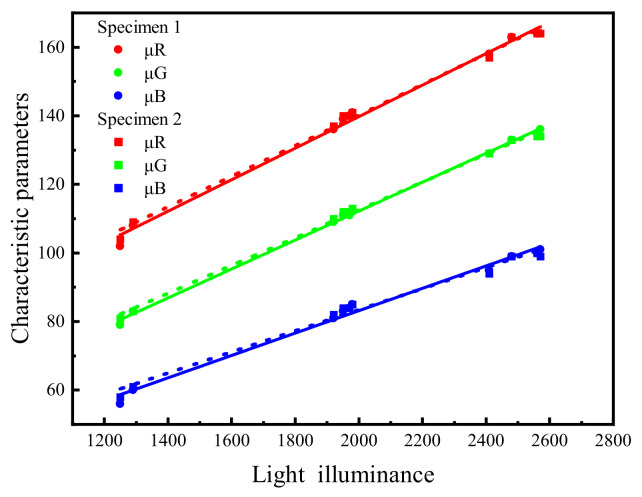
Characteristic parameters of soil image in RGB color space under different illuminance.

The relationship between the characteristic parameters from the RGB color space for specimen image and illuminance is shown in Figure 9. The characteristic parameters increased with the increase in illuminance, and there was an obvious linear relationship (R^2^ ≥ 0.98), which is consistent with Han et al. [21]. This means that the characteristic parameters of soil image under different illuminance could be converted to that under the reference illuminance.

**Figure 10:**
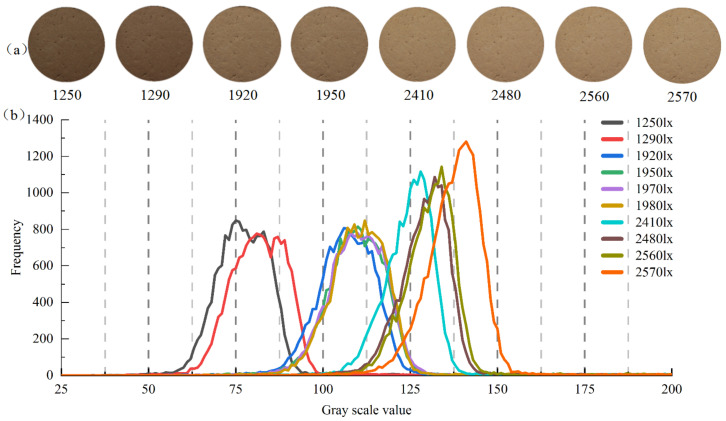
Image and gray scale curve under different illuminance: (**a**) original soil sample images; (**b**) image gray histograms.

The relationship between the gray histogram of image and the specimen surface illumination is shown in Figure 10. It shows that with the increase in illumination, the brightness of image increased. The 10 gray histograms could be roughly divided into three groups: group I (1250~1290 lx), group II (1920~1980 lx), and group III (2410~2570 lx). The gray levels of three groups were 50~100, 75~125, and 100~165, respectively, and the frequency peaks were about 800, 800, and 1100, respectively. The gray histogram for the first two groups is on the left side of the diagram, their frequency peaks are close to each other, and images are dark. The frequency peaks and kurtosis of group III changed more sharply compared with groups I and II. When the illuminance was less than 1900 lx, for the soil specimens with high water content or dark color, the gray histogram of their images continued to move left, and the gray level interval that could be used to describe the variation of water content was narrowed. When the illuminance was greater than 2500 lx, for soil specimens with smooth surface or water film, the collected image was prone to distortion due to overexposure, its gray histogram shifted right, and the frequency peak and kurtosis increased significantly. Meanwhile, the effective color information including water content in the image was reduced. In short, if the illuminance was selected in the range of 1900~2500 lx, the gray histogram of soil specimen image distributed in the middle. This illuminance range could cover a wider range of water content change, so it could be used as optimal surface illuminance for soil color acquisition. However, the optimal illuminance was related to light source type, image acquisition distance, and soil type. 

### 3.3. Shooting Angle and Ambient Temperature

#### 3.3.1. Shooting Angle

**Figure 11:**
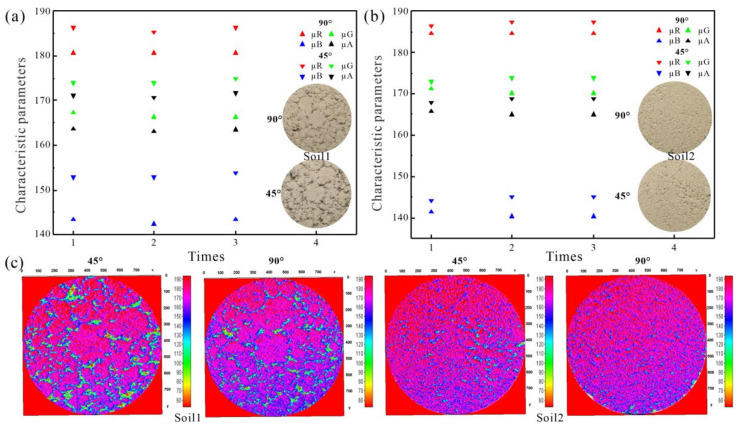
Characteristic parameters in RGB color space under different shooting angles: (**a**) and (**b**) characteristic parameters of Soil 1 and Soil 2 respectively derived from the RGB color space; (**c**) distribution of surface gray scale values.

The characteristic parameters and surface gray level of calibration soil specimen are shown in Figure 11. The characteristic parameters of the soil image at a shooting angle of 45° were larger than that at 90°. The reason is that the visible spectrum signal collected by the camera at the former angle is mainly derived from the specular reflection of soil surface while that at the latter angle is derived from the diffuse reflection of soil surface, so the former reflection signal is stronger than the latter [43]. Figure 11 also shows that the soil specimen image collected at a 45° shooting angle contained more unnecessary information such as shadows and holes on the soil specimen surface, which affected the color authenticity and interfered with the subsequent image processing.

**Figure 12:**
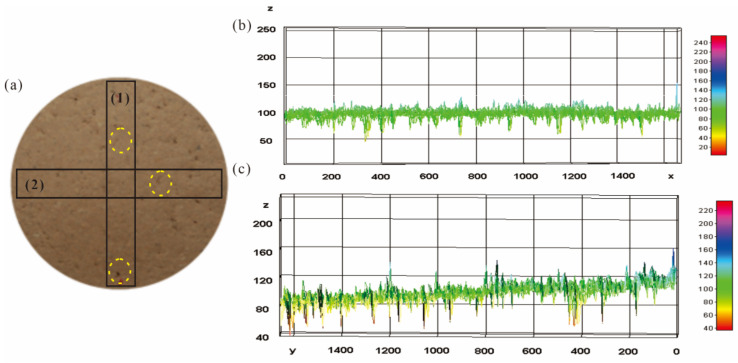
Soil image and color spatial distribution: (**a**) original soil image; (**b**) gray level of rectangle 2; (**c**) gray level of rectangle 1.

The color spatial distribution of soil specimen at a 90° shooting angle is shown in Figure 12. Extracting gray levels along the longitudinal rectangle (rectangle 1) and transverse rectangle (rectangle 2) from the original image, the gray level curves could be obtained as shown in Figure 12b,c. Both curves had local fluctuations caused by surface micro-topography due to sample preparation. Curves in Figure 12b were almost horizontal, and those in Figure 12c rose slowly. The closer the region was to the top of soil specimen, the higher the gray level was. The reason for this uneven brightness distribution is that the distance between the light source and the soil specimen was different. However, this can be corrected or eliminated by selecting an appropriate image representation area and extracting characteristic parameters with statistical significance. According to the above-mentioned analysis, a 90° shooting angle was more suitable for image acquisition of soil. Since the incident angle of the light source and the shooting angle of an image have a significant influence on the reflectivity received by a camera and the anisotropy of reflectivity may contain more water content information of a soil [44,45], the relationship between characteristic parameters and water content under different shooting angles should be explored further.

#### 3.3.2. Ambient Temperature

The statistical results of characteristic parameters from the RGB color space, as shown in Table 3, varied with ambient temperature (*T*/°C). Overall, the characteristic parameters did not change significantly with temperature. When the range of temperature reached 12.2 °C, the varying range of characteristic parameters (*μ_R_*, *μ_G_*, and *μ_B_*) was about six. If the temperature variation of ambient was less than 5 °C, the change of characteristic parameters of the same soil specimen would be limited within about ±2 (the corresponding water content fluctuated about 0.5~1.0%). If the variation of ambient temperature further increased, the image sensor of digital camera would also produce more thermal noise and reduce the signal-to-noise ratio of an image [46]. In this case, temperature correction should be considered in the image process.

### 3.4. Response of Characteristic Parameters to Water Content

**Figure 13:**
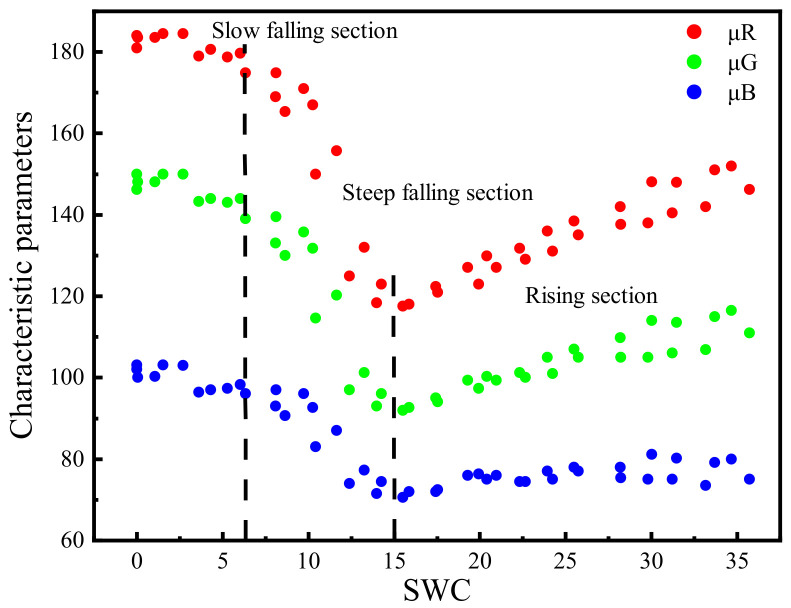
Curves of relationships between characteristic parameters from RGB color space and SWC.

The relationship between the characteristic parameters from the RGB color space and water content is shown in Figure 13. The overall trend of the curve was to descend first then ascend, consistent with the results in the literature [13,47,48]. The curve could be roughly divided into a falling section and a rising section. When water content *θ* was less than 15%, the curve was in falling section, which could be further subdivided into a slow falling section (*θ* < 6%) and a steep falling section (*θ* = 6–15%), and *μ_R_*, *μ_G_*, and *μ_B_* decreased first slowly then rapidly with the increase in water content. When *θ* was greater than 15%, *μ_R_*, *μ_G_*, and *μ_B_* increased with the increasing water content, called a rising section.

**Figure 14:**
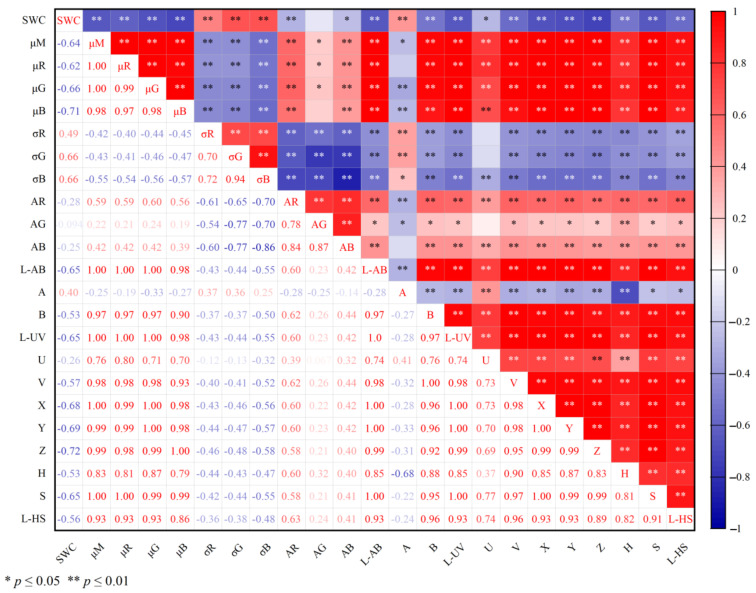
Correlation coefficient matrix between characteristic parameters and overall SWC (* and ** indicate significance at the 0.05, 0.01 level respectively; the colored numbers indicate the correlation coefficient between any two characteristic parameters; negative correlation is shown by the blue color, whereas positive correlation is indicated by the red color).

Different parameters from color spaces have different angles to represent soil color information, and their capabilities to predict water content are also different. Parameters with a strong correlation to water content can be selected by correlation analysis. The correlation coefficient matrix between 21 characteristic parameters and overall water content is shown in Figure 14. Except *A_G_*, *A_B_*, and *U*, the correlation between other characteristic parameters and water content reached a significant level (*p* < 0.01) and was mainly negative, indicating that the color information of soil digital image had the potential to predict water content. For color spaces, the characteristic parameters of CIEXYZ and RGB color spaces had the strongest correlation with water content, which were −0.68(*X*), −0.69(*Y*), and −0.72(*Z*) and −0.62(*μ_R_*), −0.62(*μ_G_*), and −0.71(*μ_B_*), respectively, but the former had lower self-correlation. For the 21 characteristic parameters, the correlation between brightness and water content was more significant, which was obviously better than that between parameters of hue, saturation, auxiliary, and water content.

**Figure 15:**
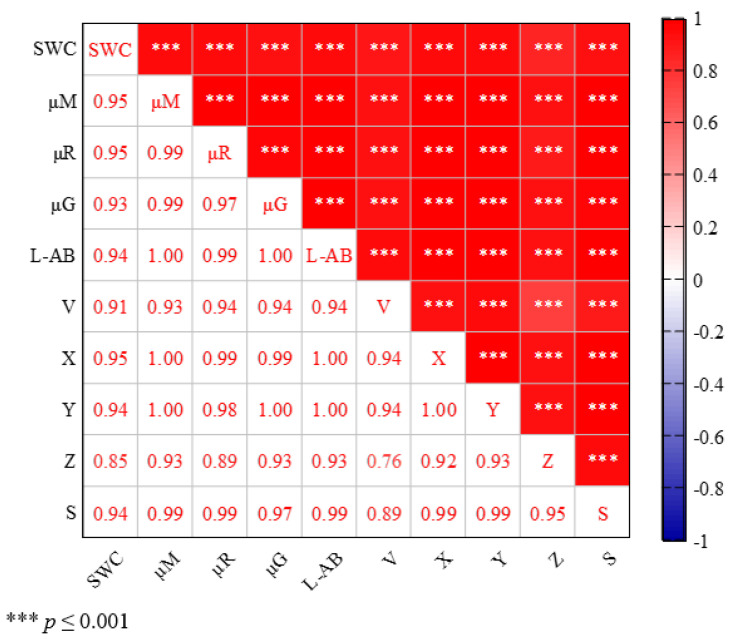
Correlation coefficient matrix between characteristic parameters and SWC of the rising section (*** indicate significance at the 0.001 level respectively; the colored numbers indicate the correlation coefficient between any two characteristic parameters; negative correlation is shown by the blue color, whereas positive correlation is indicated by the red color).

The correlation coefficient matrix related to the curves of the rising section in Figure 12 was analyzed separately, as shown in Figure 15 (the correlation coefficient lower than 0.85 is not given). Likewise, the correlation between the characteristic parameters of CIEXYZ and RGB color spaces and the water content was still the strongest but the significance level was further improved (*p* < 0.001), so the piecewise modeling method was more reasonable.

### 3.5. Evaluation of Model Accuracy

**Figure 16:**
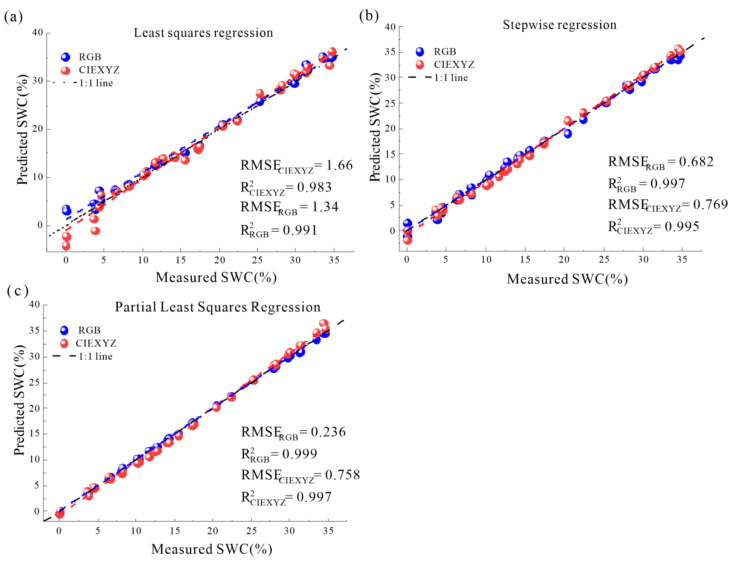
Validation results of different models of SWC prediction: (**a**) the LSR model; (**b**) the STR model; (**c**) the PLSR model.

According to the correlation analysis, six main characteristic parameters (correlation coefficient greater than 0.8) and six auxiliary characteristic parameters from RGB and CIEXYZ color spaces could be selected as input variables. A piecewise prediction model for water content was built by using LSR, STR, and PLSR. The prediction results of models are shown in Table 4 and Figure 16. Overall, the six prediction models produced R^2^ values ranging from 0.983 to 0.999, and the RMSE values ranged from 1.66 to 0.236, confirming that the piecewise linear models had outstanding prediction capacities. Because of close values of R^2^ for the models, only RMSE could be used as a valid evaluation indicator. The models using characteristic parameters in the RGB color space with generally lower RMSE produced better results than those in the CIEXYZ color space. The results demonstrated that the RGB color space was most suitable for training the SWC prediction models for the studied soil. In contrast, the prediction accuracy of the LSR model was the worst (RMSE > 1.3). This may be attributed to the loss of discriminative information by using only one channel as the input parameter of the model. The STR model produced better prediction after introducing more input variables. The RMSE of the STR model was reduced to half of that of the LSR model. The PLSR model using soil images ID represented the lowest dispersion (RMSE = 0.236) between the measured and predicted values of SWC, closely following the 1:1 line (Figure 16). As can be seen from Figure 16a,b, the prediction accuracy of the model in the slow falling section was low, which may be due to the insignificant change of reflectivity. Compared with other similar methods [12,13,49], the prediction error of models in this paper was small. The reason is that in this experiment, we strictly controlled the quality of the soil images, extracted the characteristic parameters with statistical significance by Gaussian fitting, and considered piecewise modeling, which significantly reduced the interference of external factors on soil image acquisition and data processing error. As the image sensor of the camera could only receive visible spectrum (400~700 nm) signals, there is still a gap in the prediction accuracy compared with the visible and near-infrared spectral (400~2500 nm) methods [50]. However, this deficiency can be further compensated by expanding the database and introducing a machine learning algorithm.

Despite extensive studies on soil color and its properties, the quantitative link between SWC and soil color is still inadequately comprehended. This paper highlighted the features and advantages of combining equipment for collecting high-quality soil images with piecewise models for non-destructive SWC prediction. Nevertheless, there were several associated limitations for practical applications. First of all, it is difficult to implement field applications due to the limitation of light source conditions for soil image acquisition. Moreover, in order to simplify the influencing factors and eliminate the interference of impurities in soil (such as roots and loess solidification), the surface soil was removed. The approach for soil color in SWC prediction has to be investigated in depth for soils with complex impurities so that it may be used more effectively in agriculture. In addition, this method may only be appropriate for the same type of soil with a similar composition of minerals and particles. More studies should focus on different soils. Despite the existing constraints on experimental settings and soil samples, it was verified in this paper that rapid, non-destructive, and accurate SWC prediction can be performed in a controlled condition.

This study was only a preliminary exploration. In a subsequent stage, the pre-trained color water content model may be employed in a laboratory evaporation experiment of expansive soil. The dynamic variations of surface water content can be obtained by recording continuous soil images, which can provide not only potential technical support for revealing the evaporation law but also necessary data support for understanding the relationship between crack development and water content.

## 4. Conclusions

In this study, the influence of light sources, light intensities, ambient temperature, and shooting angles on acquisition of stable and high-quality digital images for SWC testing was investigated. The characteristic parameters with statistical significance were extracted. The correlation between characteristic parameters of color spaces and water content was analyzed, then a related regression model for SWC prediction was developed. The following conclusions could be drawn:

(1) D65 standard light source with incident angle of 45°, image shooting angle of 90°, surface illumination of soil specimen of 1900~2500 lx, temperature variation within 5 °C, and other test parameters were comprehensively determined. The determinations could effectively overcome the distortion of visible spectrum signals in acquisition so as to obtain soil digital images with high reproducibility and stability.
(2) The image sampling area had a great impact on the extracted characteristic parameters. An optimal area could be determined for a soil image by analyzing the change of the mean and variance of color under different sampling areas. In this study, the whole image (3,000,000 pixels) was chosen for Gaussian fitting to obtain the characteristic parameters with statistical significance, which could weaken the influence of spatial variation of image color and the surface micro-topography, but the anti-interference performance of the parameters was significantly better than that of the mean and median values.
(3) Within the tested range of water content, the lightness of CIEXYZ and RGB color spaces had the strongest correlation with the water content of soil specimen among the 21 characteristic parameters of five color spaces; and *X*, *Y*, *Z*, *μ_R_*, *μ_G_*, and *μ_B_* had a significant correlation with water content. If the relationship between SWC and characteristic parameters is considered in sections, the significance level between them can be further improved.
(4) SWC prediction model based on the RGB color space had the highest accuracy, and the PLSR model based on soil specimen image IDs was the best, which was most suitable for SWC prediction.
(5) A new exploration was performed to improve the methods of soil digital image acquisition and color information extraction, but only an expansive soil was tested in this study. More research on SWC prediction model by color of digital images for different soils and particle sizes is needed.

## Figures and Tables

**Table 1:** A summary of different methods to measure SWC.

Methods	Advantages	Drawbacks
Oven-dried method	Accurate, simple	Time-consuming, destructive, labor-intensive
TDR	Easy, applicable for fields and model tests	Complex operation, vulnerable to salt and cracks
Neutron meter	Broad range of measurements, reliable accuracy	Radioactive hazardous
Remote sensing method	Dynamic change monitoring	Heavily dependent on climate, vegetation cover

**Table 2:** Basic parameters of Canon 5D Mark Ⅱ.

Effective Megapixels (pixel)	Sensor Size (mm)	Focal Length (mm)	Shutter Speed (s)	Aperture Value	ISO	Picture Style
5616 × 3744	CMOS (35.8 × 23.9)	105	1/100	f/4	200	Reliable

**Table 3:** Characteristic parameters at different temperatures.

Parameter	Min	Max	Mean	SD	Cv(%)	Skewness
*T*	17.60	29.80	23.36	4.08	14.78	0.18
*μ_R_*	179.70	185.70	182.94	1.31	0.72	0.52
*μ_G_*	164.40	170.30	167.69	1.33	0.79	0.99
*μ_B_*	131.80	139.50	137.04	2.01	1.47	1.29
*μ_A_*	158.63	164.70	162.65	1.38	0.85	1.69

**Table 4:** Evaluation of different SWC predictive models.

Model	Threshold Value	Validation Sets					
		RGB			CIEXYZ		
		Variables or Formula	R^2^	RMSE	Variables or Formula	R^2^	RMSE
LSR	*θ* < 6%	*θ* = −0.581 × *μ_M_* + 93.696	0.991	1.345	*θ* = −1.218 × *X* + 43.178	0.983	1.668
	6% < *θ* < 15%	*θ* = −0.136 × *μ_M_* + 28.018			*θ* = −0.395 × *X* + 18.991		
	*θ* > 15%,	*θ* = 0.701 × *μ_M_* − 56.308			*θ* = 2.251 × *X* − 12.521		
STR	*θ* < 6%	*θ* = −52.208 − 0.389 × *μ_R_* − 0.134 × *μ_G_* + 7.137 × *σ_G_* + 1847.125 × *A_G_*	0.997	0.682	*θ* = 34.559 − 0.026 × *X* − 1.943 × *Z*	0.995	0.769
	6% < *θ* < 15%	*θ* = 44.212 − 0.119 × *μ_R_* + 0.015 × *μ_G_* − 0.569 × *σ_G_* − 320.140 × *A_G_*			*θ* = 15.584 − 0.972 × *X* + 1.351 × *Z*		
	*θ* > 15%,	*θ* = −100.081 + 0.239 × *μ_R_* − 0.112 × *μ_G_* + 3.281 × *σ_G_* + 1067.108 × *A_G_*			*θ* = −8.723 + 2.621 × *X* − 1.094 × Z		
PLSR	*θ* < 6%	*μ_R_*, *μ_G_*, *μ_B_*, *σ_R_*, *σ_G_*, *σ_B_*, *A_R_*, *A_G_*, *A_B_*	0.999	0.236	*X*, *Y*, *Z*	0.997	0.758
	6% < *θ* < 15%						
	*θ* > 15%						

## Data Availability

Not applicable.
